# Current and projected burden of heart failure in the Australian adult population: a substantive but still ill-defined major health issue

**DOI:** 10.1186/s12913-016-1748-0

**Published:** 2016-09-21

**Authors:** Yih-Kai Chan, Camilla Tuttle, Jocasta Ball, Tiew-Hwa Katherine Teng, Yasmin Ahamed, Melinda Jane Carrington, Simon Stewart

**Affiliations:** 1 Mary MacKillop Institute for Health Research, Australian Catholic University, Level 5, 215 Spring Street, Melbourne, VIC 3000 Australia; 2 Baker IDI Central Australia, Alice Springs, Northern Territory 0870 Australia; 3 Western Australian Centre for Rural Health, University of Western Australia, Perth, Australia

**Keywords:** Heart failure, Prevalence, Incidence, Economic burden

## Abstract

**Background:**

Comprehensive epidemiological data to describe the burden of heart failure (HF) in Australia remain lacking despite its importance as a major health issue. Herewith, we estimate the current and future burden of HF in Australia using best available data.

**Methods:**

Australian-specific and the most congruent international epidemiological and health utilisation data were applied to the Australian population (adults aged ≥ 45 years, 8.9 of 22.7 million total population in 2014) on an age and sex-specific basis. We estimated the current incident and prevalent cases of clinically overt/symptomatic HF (predominately those with reduced ejection fraction), hospital activity (diagnosis of HF as a primary or secondary reason for admission) and health care costs in 2014 and future prevalence and burden of HF projected to 2030.

**Results:**

We estimated that over 61,000 (6.9 per 1000 person-years) adult Australians aged ≥ 45 years (58 % women) are diagnosed with HF with clinically overt signs and symptoms every year. On a conservative basis, 480,000 (6.3 %, 95 % CI 2.6 to 10.0 %) Australians (66 % men) are now affected by the syndrome with > 150,000 hospitalisations in excess of 1 million days in hospital per annum. The annual cost of managing HF in the community is approximately $900 million and nearly $2.7 billion ($1.5 versus $1.2 billion, men versus women) when considering the additional cost of in-patient care. We predict that the prevalence and future burden of HF will continue to increase over the next 10–15 years to nearly 750,000 people with an estimated annual health care cost of $3.8 billion.

**Conclusions:**

Australia is not immune to the growing magnitude and implications of a sustained epidemic of HF in an ageing population. However, its public health and economic burden will remain ill-defined until more definitive Australian-specific data are generated.

## Background

Heart failure (HF) is one of the most prevalent cardiovascular diseases worldwide and is routinely attributed to be the leading cause of hospitalisation in persons aged ≥ 65 years [1]. Despite a relative paucity of specific information (from its epidemiology to health care episodes), Australia is not immune to this significant public health issue. More than a decade ago, we estimated that approximately 325,000 adult Australians (4.5 % of those aged ≥ 45 years) were directly affected by this complex syndrome with around 100,000 hospital admissions per annum attributable to HF overall [2]. Ominously, for the Australian health care system, we also identified around 214,000 Australians with asymptomatic left ventricular systolic dysfunction at that time and have tracked residually high levels of antecedent risk for developing the syndrome; particularly relating to remnant high blood pressure levels in the community [3] and suboptimal levels of hypertension management in primary care [4]. At the same time, the Australian population has not only expanded but progressively aged since our last HF burden estimates. The latter becomes an increasingly important factor when considering the scope of HF has evolved with increasing awareness and recognition of HF associated with preserved ejection fraction (HFpEF - particularly among older women with a history of hypertension) [5]. This clinical entity remains problematic both in terms of diagnosis and treatment [6, 7]. In this context, HF with reduced ejection fraction (HFrEF) [6] remains a major clinical and public health focus with the efforts to improve its detection and treatment continuously evolving.

Importantly, since our last set of estimates (largely reliant on international data), a number of recent Australian-specific studies relating to the population prevalence of HF (notably the Canberra Heart Study [8]) and HF-related hospital activity (the West Australian linked data resource [9]) have provided a greater certainty around the epidemiological profiling of the syndrome when extrapolated to the latest and projected population figures for the whole of Australia [10, 11]. For the purpose of this study, we largely focused on incident and prevalent cases of HFrEF with or without a component of diastolic dysfunction (the hallmark of HFpEF) within the Australian population whilst providing some estimates of the likely burden imposed by HFpEF alone.

### Study aims

Based on the changing population dynamics and more Australian-specific data, we aimed to produce a more accurate set of figures (from its population profile to hospital and community care activity) to describe various aspects of the contemporary burden of HF (as noted, predominantly that associated with systolic dysfunction as evidenced by reduced left ventricular ejection fraction) in Australia.

## Methods

All data sources had appropriate ethics approval and this study was conducted according to the principles outlined in the Declaration of Helsinki [12].

### Investigational strategy and data sources

To conservatively estimate the current incident and prevalent cases of HFrEF in the Australian adult population (aged ≥ 45 years), we evaluated a combination of validated Australian-specific and international peer-reviewed epidemiological and clinical trial datasets (see Table 1). These were applied to the latest Australian Bureau of Statistics population figures according to geographic locale on an age and sex-specific basis [10]. Based on our previously published reports [2, 13], this represents a validated method for estimating the burden of HF in Australia [2] and beyond [13]. Consistent with this approach, the following were applied when selecting data to shape our burden estimates: 1) original Australian data (via a systematic review of the literature and in consultation with a panel of Australian HF clinical research academics/experts) were utilised [8, 9] in preference to overseas data; 2) preference was given to the most comprehensive and contemporary datasets or according to the purpose it was best suited, this included use of Western Australia linked data [9] to estimate new/*de novo* HF-related admissions as opposed to the broader New South Wales data [14] describing all primary and secondary admissions for HF per annum; and 3) where there were no contemporary Australian-specific data available, the most congruent international data were identified and utilised [15–17].

**Table 1:** Datasets – purpose and references

Datasets	Purpose	Reference
Population profile		
- Australian Demographics Statistics 2014	To obtain data representating the Australian population by sex and age as calculated on 30 June 2014	ABS [10]
Incident cases		
- Incidence and aetiology of heart failure; a population-based study	To determine the population incidence of heart failure data by sex and age groups	Cowie et al. [15]
- Quantifying the heart failure epidemic: prevalence, incidence rate, lifetime risk and prognosis of heart failure: The Rotterdam Study		Bleumink et al. [16]
Prevalent cases		
- Congestive heart failure in the community a study of all incident cases in Olmsted County, Minnesota in 1991	To determine the population prevalence of heart failure data by sex and age groups	Senni et al. [17]
- Prevalence of heart failure and systolic ventricular dysfunction in older Australians: the Canberra Heart Study		Abhayaratna et al. [8]
- Quantifying the heart failure epidemic: prevalence, incidence rate, lifetime risk and prognosis of HF: The Rotterdam Study		Bleumink et al. [16]
Hospital activity		
- Heart Failure Incidence, Case Fatality, and Hospitalisation Rates in Western Australia Between 1990 and 2005	To evaluate the hospital burden of heart failure	Teng et al. [9]
Cost and burden of HF		
- Pressure points in primary care: blood pressure and management of hypertension in 532,050 patients from 2005 to 2010	To investigate the primary care burden of heart failure	Carrington et al. [4]
- Impact of Home Versus Clinic-Based Management of Chronic Heart failure The WHICH? Multicenter, Randomized Trial	To calculate the financial burden of heart failure	Stewart et al. [18]
Future projection		
- Population Projections, Australia, 2012 (base) to 2101	To calculate the projection data to 2030	ABS [11]

### Population profile

We obtained the Australian Bureau of Statistics Australian population data on an age and sex-specific basis and according to geographic locale as at June 2014 [10]. Data for all persons aged ≥ 45 years (8,864,528) were grouped into 10-year age brackets except for those aged ≥ 75 years, who were treated as a single group. This was undertaken for both men and women and for each Australian State and Territory.

### Incident and prevalent cases

Incident cases were defined as the annual number of new/*de novo* cases of HF predominantly associated with HFrEF (where individuals must present with appropriate symptoms and anomalies in the underlying cardiac structure and function associated with systolic dysfunction as evidenced by reduced left ventricular ejection fraction) and calculated by applying annual age and sex-specific incidence rates derived from international incidence statistics [15, 16] to the Australian Bureau of Statistics 2014 Australian population by 10-year age groups. Similarly, prevalent cases were defined as the combined total of new/*de novo* and surviving/pre-existing cases of HFrEF and calculated based on an annual point prevalence basis using a combination of Australian [8] and international [16, 17] prevalence data.

### Hospital activity

A broad range of parameters pertaining to HF-related hospitalisations (confirmed by the International Classification of Diseases 9^th^ edition [ICD-9] and 10^th^ edition [ICD-10] diagnostic codes for HF) were derived from Australian-specific data alone [9]. This includes estimates of: 1) incident hospital admissions associated with a primary (ICD-9 codes: 428x, 402.01, 402.11, 402.91, 404.1, 404.3, 425x, 518.4, 514, 391.8, and 398.91, and ICD-10 codes: I50x, I11.0, I13.0, I13.2, I42x, J81, I01.8, I02.0) or secondary (with a principal diagnosis of a cardiovascular condition such as ischemic heart disease or atrial fibrillation, but not acute myocardial infarction) diagnosis of composite HF, 2) type of admission (unplanned or planned), 3) length of hospital stay (LOS), 4) in-patient case-fatality, 5) discharge destinations (i.e. own home, acute hospital or long-term rehabilitation or residential/supported care), 6) readmissions within 12 months and 7) any hospital admissions (new/*de novo* or recurrent event) associated with a primary or secondary diagnosis of HF overall and LOS per annum.

### Health care costs

Estimations of the annual cost of managing those individuals hospitalised with HF including the cost of their in-patient care (including *per diem* hospital costs) and auxiliary device therapy plus associated community management costs were based on a recently published HF-specific management trial detailing all health care costs typically associated with the management/care of patients presented with a composite diagnosis of HF within Australia [18]. These were applied to prevalent cases of HF (but not additional cases of HFpEF alone). Specifically, community management costs including allied health professionals and health services respective unit cost ($1825 per year) was multiplied by all HFrEF cases with an adjustment for days alive and out-of-hospital (99.4 %). All HF-related hospital episodes were multiplied by the average cost of hospital stay ($1806 per day).

### Future burden

The estimated future trend and growth rate in incidence and prevalence of HF (once again predominantly that associated with HFrEF) was projected to 2030 using the latest population projection data (released in 2013) that included a moderate assumption on future fertility and mortality rates and a constant net migration [11]. Conservatively, we assumed that the incident and prevalent cases of HF would remain stable from 2014 to 2030 and the same occurrence statistics were applied to an increasingly ageing Australian population to form the projection data.

### Population prevalence of HFpEF

In order to estimate the additional contribution of those individuals with HFpEF alone (with clear expectations of older, more female cases with a history of hypertension), we applied international HFpEF prevalence statistics [17] according to age and sex, to the Australian population by 10-year age groups to calculate the point prevalence of HFpEF as at June 2014.

## Results

### Incident cases

**Fig. 1:**
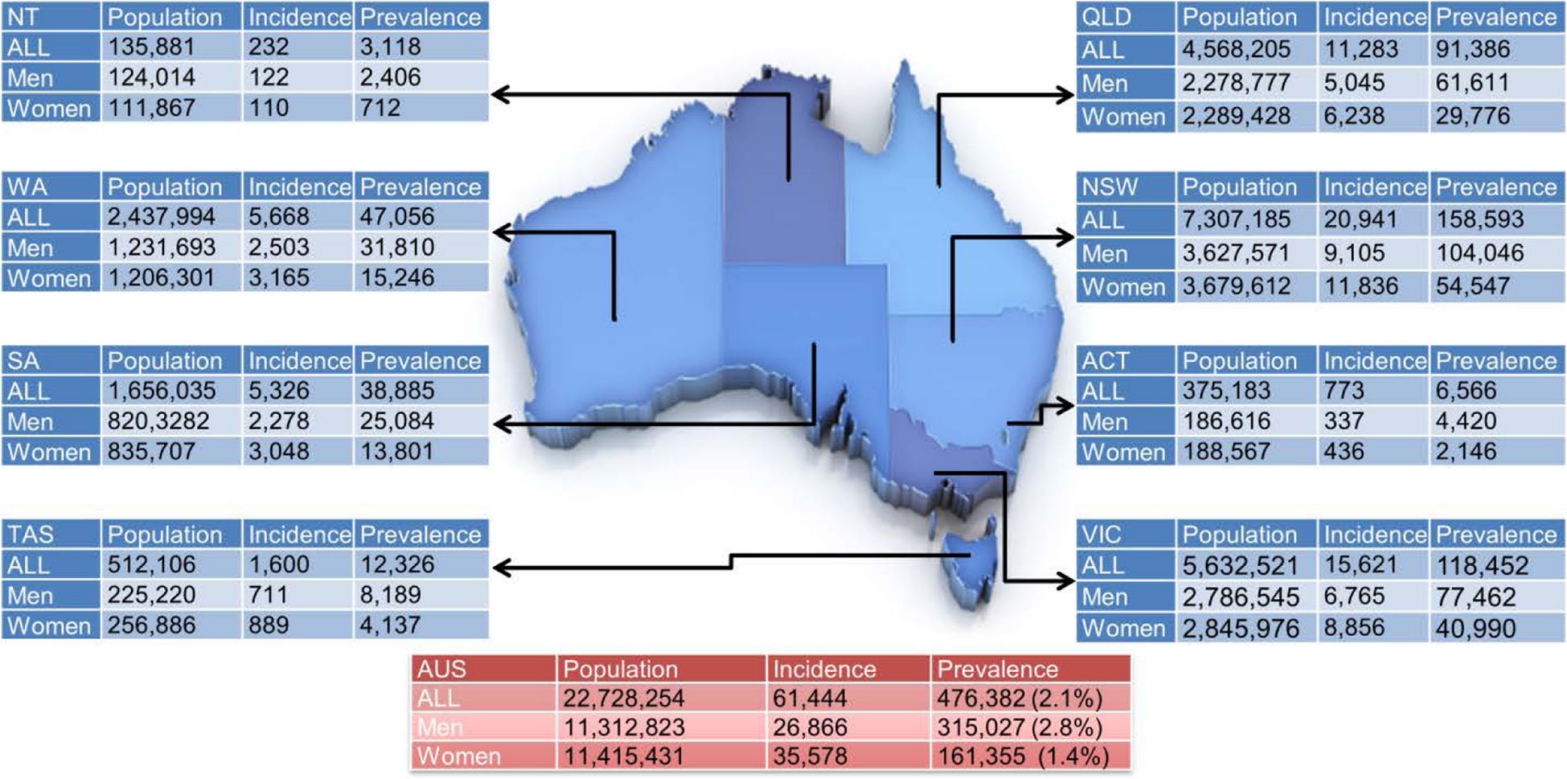
Incident and prevalent cases of heart failure with reduced ejection fraction (HFrEF) in the Australian population according to State and Territory

Annually, we estimate that over 61,000 (or 6.9 per 1000 person-years) Australians aged ≥ 45 years (58 % women) are diagnosed with HF (Fig. 1). The incidence rate is higher in men (0.3 per 1000 person-years) aged 45 to 54 years when compared to women of the same age range (0.1 per 1000 person-years), with a relatively similar upward pattern for both sexes aged 55 to 74 years. However, in the older groups, the incidence rate increases exponentially in both men and women. Greater female longevity translates into women have higher incidence rates (29.2 per 1000 person-years) in those aged ≥ 75 years (Table 2).

**Table 2:** Australian adult population (aged ≥ 45 years) and estimated incident and prevalent cases of heart failure with reduced ejection fraction (HFrEF) and all hospital activity associated with a primary or secondary composite diagnosis of heart failure

Age (years)	Population	Incident cases^a^	Prevalent cases	Hospital admissions^b^ (primary/secondary)	LOS^g^ (days)
Men					
45–54	1,513,403	454 (0.3)^c^	77,171 (5.1 %)^e^	18,732 (123.8)^g^	114,298
55–64	1,283,890	2,831 (2.2)^d^	94,992 (7.4 %)^e^	15,747 (122.7)^g^	95,191
65–74	879,090	7,085 (8.1)^d^	56,376 (6.4 %)^f^	16,802 (191.1)^g^	113,030
75+	606,842	16,498 (27.2)^d^	86,488 (14.3 %)^f^	21,363 (352.0)^g^	168,600
Total	4,283,225	26,867 (6.3)	315,027 (7.4 %)	72,644 (169.6)	491,119
Women					
45–54	1,543,002	154 (0.1)^c^	15,428 (1.0 %)^e^	18,680 (121.1)^g^	113,985
55–64	1,306,222	2,876 (2.2)^d^	28,734 (2.2 %)^e^	15,655 (119.8)^g^	94,687
65–74	899,957	7,281 (8.1)^d^	28,760 (3.2 %)^f^	14,783 (164.3)^g^	99,466
75+	832,122	24,268 (29.2)^d^	88,433 (10.6 %)^f^	25,576 (307.4)^g^	206,856
Total	4,581,303	34,580 (7.5)	161,355 (3.5 %)	74,703 (163.1)	514,994

*LOS* length of stay

^a^Cases per 1,000 person-year in the parentheses; ^b^admissions rate per 10,000 person-year in the parentheses; primary or secondary composite diagnosis of heart failure

Key statistics used for estimation ^c^Cowie et al., ^d^Bleumink et al., ^e^Senni et al., ^f^Abhayaratna et al., ^g^Teng et al.

### Prevalent cases

Overall, we estimated that, on an annual basis, approximately 480,000 Australians (66 % men) are affected by HF predominantly associated with HFrEF. This equates to 6.3 % (95 % CI 2.6 to 10.0 %) of those aged ≥ 45 years or 2.1 % (2.8 % of men and 1.4 % of women) of the entire Australian population of 22 million people in 2014. As anticipated, Fig. 1 shows that most cases were from the most populous States on the Eastern seaboard of Australia. New South Wales and the Northern Territory had the highest and lowest number of people affected by HFrEF overall with 158,593 (33 %) and 3118 (0.7 %) cases, respectively. Reflective of global patterns and the clinical paradigm of HF, the prevalence estimates are five times higher in men (77,171, 5.1 %) than in women (15,428, 1 %) aged 45 to 54 years and remain higher at each age-group from 55 to 74 years. However, there is a sharp increase in prevalent cases of HF in women aged ≥ 75 years (Table 2).

### Hospital activity

There were an estimated 27,468 (45 % of all incident cases) new HF-related incident admissions in 2014, of which 60 % were admitted with a primary diagnosis of HF. Incident admissions increase steeply with advancing age, especially as a primary diagnosis in those aged ≥ 65 years. More than 80 % of all incident HF-related admissions were ‘unplanned’ and the total annual LOS associated with these admissions was approximately 225,000 days (average 8 days per admission). The majority of those who survive the index-event return to their own home post-discharge (79 %) and the remainder (increasing with age) receive ongoing management via another acute care facility or, due to general health deterioration, require ongoing residential care and support (21 %). This latter (and costly) phenomenon becomes increasingly more likely with each repetitive hospital admission.

The total number of readmissions within the 12-months following an incident hospitalisation for HF (of any diagnosis) is estimated to be > 10,000 separations (37 % of all incident HF-related admissions). Reflective of an increasing clinical complexity, often with multimorbidity for those with the syndrome, the risk of readmission increases steeply with age especially among men aged up to 75 years (750 separations per 5-year age group) and women aged ≥ 85 years (1800 separations).

Overall, we estimated that HF contributes to > 147,000 hospital admissions (rate of 166.2 per 10,000 population). This comprises 45,000 separations (50.6 per 10,000 population) as a primary diagnosis and 102,000 separations (115.6 per 10,000 population) as a secondary diagnosis. This results in > 1 million days in hospital each year. Of these, the greatest burden of this debilitating condition is in hospital stay among persons aged ≥ 65 years (58 % or 587,952 days with a mean LOS of 7.5 days) compared to those aged < 65 years (42 % or 418,161 days with a mean LOS of 6.1 days; Table 2).

### Costs

**Fig. 2:**
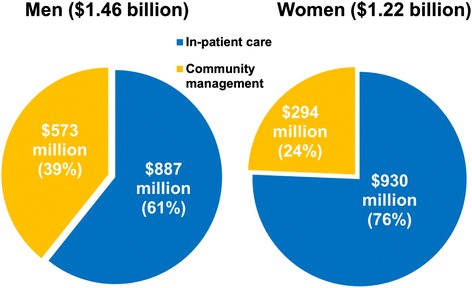
Estimated current direct health care cost of clinically overt heart failure according to sex and type of care

Based on the number of prevalent cases of HF (predominantly those with HFrEF), we estimated that the annual community management cost is approximately $867 million and nearly $2.68 billion per annum ($1.46 billion for men versus $1.22 billion for women) when considering the additional cost of in-patient care ($1.82 billion or 68 % of total expenditure) associated with all HF-related hospital admissions. Overall, the cost of in-patient care is twice as high as community care, and in women only the ratio is three times as high (Fig. 2) with readmissions constituting a substantial proportion of all hospitalisation costs.

### Future burden

**Fig. 3:**
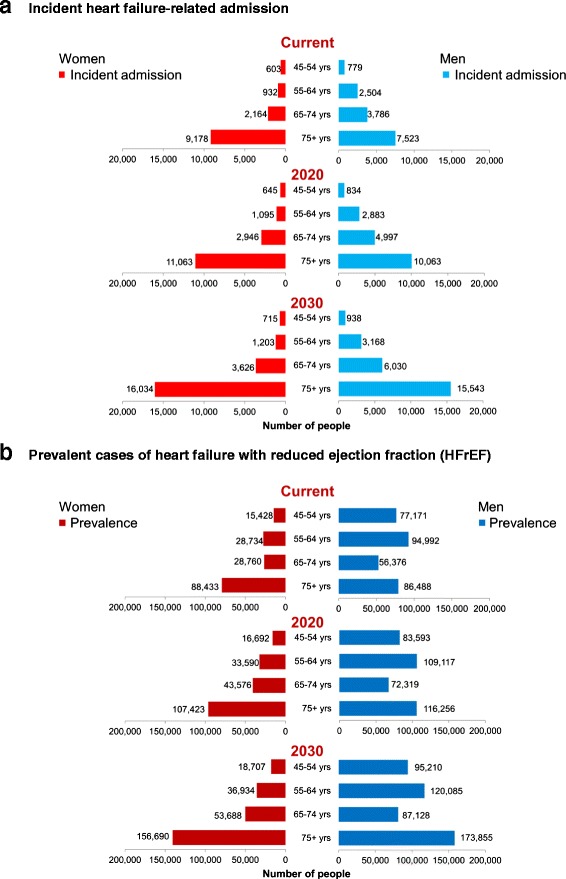
Current and projected estimations of **a** incident heart failure-related admissions and **b** overall prevalent cases of heart failure with reduced ejection fraction (HFrEF) from 2014 to 2030 according to sex and age categories

Based on a conservative assumption that incident and prevalent cases of HF (once again predominantly that relating to HFrEF) would remain stable from 2014 to 2030, we predict that the annual number of HF-related incident admissions will continue to rise to nearly 35,000 (in 2020) and to more than 47,000 by 2030 (Fig. 3). As expected, the projected increase in incident admissions in persons aged ≥ 75 years is significantly greater than in those aged ≤ 65 years (89 % versus 31 %). Consequently, we estimate that by 2020, there will be a minimum prevalent population of approximately 580,000 cases of HF (an increase of 21 % in men and 25 % in women) and as many as 750,000 Australians will be affected by this debilitating syndrome in 2030 (an increase in prevalence of 51 % in men and 65 % in women from 2014). Taken together, this represents a significant increase in an ageing and rapidly expanding Australian population even without any changes to incidence or survival rates. We also predict that the prevalence gap between men and women will continue and perhaps widen over time, potentially due to the increase in the number of older women affected by the syndrome.

### Additional population prevalence of HFpEF

**Fig. 4:**
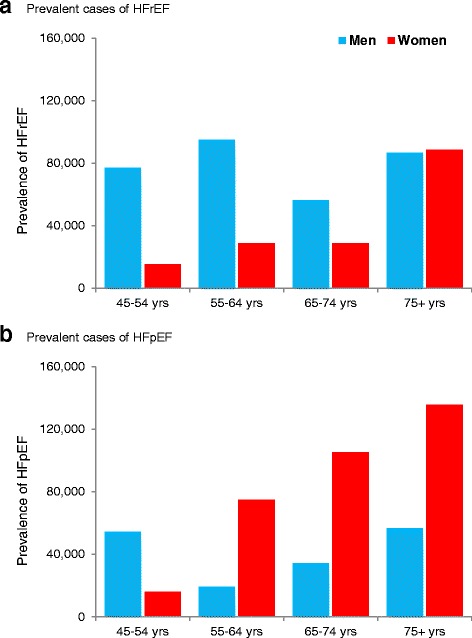
Prevalent cases of heart failure associated with **a** reduced ejection fraction (HFrEF) and **b** preserved ejection fraction (HFpEF) in Australians aged ≥ 45 years according to age categories

We estimated that an additional 496,000 Australians (67 % women) aged ≥ 45 years (6.6 %, 95 % CI 2.1 % to 11.1 %) are affected by HFpEF alone each year. In contrast to the age and sex distribution of HFrEF, more women (331,670) than men (164,182) were likely to be affected by HFpEF. With the exception of those in the younger age group (45 to 54 years) where there were three times more men (54,331) than women (15,893), there were significantly more women than men with HFpEF in successively older age groups (Fig. 4).

## Discussion

Despite wide recognition of an evolving burden of HF globally, there is a lack of high-quality clinical/epidemiological data to quantify the number of Australians affected by this deadly syndrome. Using contemporary population data and conservative estimates derived from robust Australian and international studies, we estimate that approximately 61,000 new cases of clinically overt HF are diagnosed yearly, with a prevalence approaching 480,000 and around 150,000 hospital admissions associated this syndrome overall. Collectively, patients with a composite diagnosis (either as primary or secondary) of HF probably contribute to over 1 million days in hospital at a cost of more than $2.6 billion (largely due to recurrent hospital care/episodes). We also predict that, without a substantial change in the drivers of the syndrome, prevalence of HF predominantly associated with HFrEF will continue to grow over the next 10–15 years to nearly 750,000 people with an annual managing cost in excess of $3.8 billion by 2030. These figures do not reflect the likely additional burden (predominantly affecting older women with non-ischaemic aetiology) of close to 500,000 Australians with HFpEF alone. This latter component of the HF burden remains most challenging both in terms of diagnosis and treatment [19]. However, it cannot be ignored given the ageing Australian population, even if, as some would argue, it represents a more benign condition within the spectrum of the HF syndrome [20].

### Comparison between current and previous estimates

Since our first report on the ‘hidden epidemic of HF’ published 11 years ago [2], there has been a steady decrease in coronary artery disease mortality [21] potentially due to improved medical and therapeutic management. Although the risk of experiencing a further cardiac event is not universal and varies considerably across the spectrum of survivors, it seems probable that the success in treating these cardiac conditions will increase HF prevalence now that these patients survive and live longer with multimorbidity [22]. Our prior estimates of around 325,000 Australians living with HF, 22,000 new HF cases diagnosed and 100,000 HF-related hospitalisations in 2000 [2] is in synergy with the current estimates of 480,000 Australians with HF, 27,000 incident HF-related admissions and 147,000 hospitalisations overall, an increase of 47 %, 23 % and 47 %, respectively, from over a decade ago. As such, the current estimates support our prior conclusion of a ‘HF epidemic’ in Australia and demonstrate an upward trend over time with no sign of slowing down. These data are at odds with official estimates of around 280,000 HF cases in Australia (population prevalence 1.3 %). However, such data are almost a decade old and critically flawed by the fact that they were derived by a self-reported diagnosis of HF and/or presence of peripheral oedema [23]. In addition, we found a higher prevalence of HF specifically related to HFrEF in men than in women and an exponential trajectory in the older age groups illustrating the key influence of progressive population ageing. Our data are also consistent with European, North American and Asian studies [13, 24, 25] in exploring the size of the HF burden and its consequent health and economic impacts due to high readmission rates and long durations of hospital stay, particularly in the very elderly. However, focussing on HFrEF cases alone belies the substantive contribution of HFpEF to the current and future burden of HF in Australia and beyond; particularly when one considers that most clinical studies would suggest HF predominantly affects men but epidemiological studies suggest a more even gender balance [26].

### Public health implications

HF has become a burgeoning public health problem reaching epidemic levels especially for the older age population. Currently, our estimate of half a million Australians living with HF predominantly associated with HFrEF costs the Australian health care system billions of dollars every year as well as the broader economic/societal impact on our community, family and on an individual’s quality of life. Despite this, HF remains a poorly recognised and under-appreciated burden in Australia, and the Australian health care system remains ill-prepared to detect, prevent and manage this disabling and costly syndrome; particularly in delineating between cases of HFrEF and HFpEF and diagnosing the latter in older women (see below). Our projections demonstrate a pronounced increase in clinically overt HF cases especially for the older populations in the coming decades coupled with escalating social, health and economic implications if no changes are made.

At the same time, the definition of HFpEF has evolved and is increasingly recognised as a significant public health problem worldwide [20, 27]. However, due to the lack of Australian-specific data and potential disparities in standardised HFpEF diagnosis between different studies [5], an accurate assessment is difficult to make compared with equivalent HFrEF estimations. Individuals with HFpEF tend to be older at the time of initial diagnosis and most have a history of hypertension and/or atrial fibrillation [26, 28]. In regards to frequency in the population, we believe that HFpEF may be as prevalent as HFrEF and we estimate that women outnumber men by a 2:1 ratio and its overall prevalence among all persons with HF (HFrEF and HFpEF) was 43 % in those aged < 65 years and 56 % in those aged ≥ 65 years. Hence, it is likely that the overall mortality rate attributable to HFpEF is higher than HFrEF given the higher proportion of HFpEF in the older population.

Despite many efforts in improving quality of life and survival, HF has a poor prognosis with 12 % mortality within 30 days following an incident admission and cumulative mortality of approximately 31 % and 50 % at 1-year and 5-years, respectively. This is worse than the prognosis for most cancers [29]. It is prudent that there is an increasing focus on HF prevention rather than spending more money on expensive and less effective treatments for the syndrome. For high-risk individuals including those with hypertension, diabetes, chronic kidney disease, coronary artery disease and vascular disease, renewed efforts to prevent progressive cardiac dysfunction should be the focus of research efforts and preventative health care programs. In addition, the application and optimisation of proven strategies for HF management such as ‘gold-standard’ therapeutics [30], devices [31], tele-monitoring [32] and nurse-led multidisciplinary programs of care [33] can cost-effectively improve outcomes in HF. More efforts are also needed to gain better insight into the drivers of HF hospitalisations (often costly and prolonged) and preventable (repeated) readmissions that are imperative for improving individual care and addressing broader economic resource implications.

### Limitations

A number of specific limitations need to be reinforced in relation to the estimates derived from this study. Firstly, there is still a paucity of local data to accurately quantify the incident to prevalent cases of HF in Australia. Therefore, large-scale, population-based studies are required to ascertain the true burden of HF from a number of perspectives including health care utilisation, economic (direct and indirect) costs and it’s broader societal impact. Secondly, increasing disease awareness (including the introduction of broad screening programs), and the continuous rise in the ageing population will increase the annual incidence of HF. Thus, future projections must be interpreted with some caution, given that certain factors may negatively or positively affect HF incidence and survival rates (fundamental drivers of prevalence). After careful consideration we have not included formal sensitivity analyses (over and above providing confidence intervals for key estimates and considering key population variables in deriving future projections of HF) as per original report given the greater availability of Australian-specific data and our consideration of the additional burden imposed by HFpEF. Thirdly, the current impact of antecedents and comorbidities of HF including coronary heart disease and hypertension and the prevalence of cardiovascular disease risk factors such as diabetes, tobacco smoking, excess alcohol, obesity and physical inactivity will remain influential. These may also change over time owing to increasing prevalence or improved therapies and management. Consequently, our projections may potentially under- or over-estimate prevalence, depending on the factors considered. Finally, other confounding factors such as socioeconomic status, access to primary health and acute care services, admission thresholds for HF and climatic factors are also likely to impact on the precise estimation/prediction of the magnitude and implications of the HF burden.

## Conclusions

In summary, our report uses the latest national and international clinical and epidemiological data to generate a contemporary snapshot of the current and potential future impact of clinically overt HF in Australia. They support the expectation that HF will continue to impose a significant burden both locally and globally in the coming decades. Without a dramatic change, older and sicker Australians will develop this deadly and disabling syndrome. In response, we need clear preventative strategies to target the antecedent risk factors and broader determinants to address the complex causes of HF. In addition, we need more systematic applications that integrate cost-effective management and treatment. HF, whether it be described within the confines of HFrEF or broadened to include cases of HFpEF is an enormous detrimental public health problem now and for the foreseeable future.
